# Gastrointestinal features of pediatric iga vasculitis and their association with renal complications: an observational study

**DOI:** 10.1007/s00431-025-06157-x

**Published:** 2025-05-01

**Authors:** Mukaddes Kiliç Sağlam, Sema Yıldırım, Müferet Ergüven, Mehmet Ali Sungur

**Affiliations:** 1 Department of Pediatrics, Düzce University Faculty of Medicine Hospital Konuralp, 81010 Düzce, Turkey; 2 Department of Pediatrics, İstanbul Göztepe Prof. Dr. Süleyman Yalçın City Hospital, Istanbul, Turkey; 3 Department of Pediatric, Rheumatology, Düzce University Faculty of Medicine Hospital Konuralp, Düzce, Turkey; 4 Department of Biostatistics, Düzce University Faculty of Medicine Hospital Konuralp, Düzce, Turkey

**Keywords:** Gastrointestinal system, Henoch-Schönlein Purpura, IgA Vasculitis, Pediatric, Renal involvement

## Abstract

Immunoglobulin A (IgA) vasculitis is the most common systemic vasculitis in childhood, primarily affecting the skin, gastrointestinal system (GIS), joints, and kidneys. This study aimed to evaluate the clinical and laboratory characteristics of mild and severe GIS involvement in pediatric patients with IgA vasculitis and to investigate its association with renal involvement. A retrospective review was conducted on 794 pediatric patients diagnosed with IgA vasculitis between 1997 and 2024. Demographic data, clinical findings, and laboratory parameters were collected from patient records. GIS involvement was classified as mild (abdominal pain, vomiting, or occult blood in stool) or severe (melena, hematochezia, or intussusception). Renal involvement was defined based on hematuria, proteinuria, hypertension, or renal insufficiency. Among 794 patients, 430 (54.2%) were male, with a mean age at diagnosis of 7.8 ± 3.3 years. GIS involvement was observed in 422 (53.1%) patients, of whom 333 (78.9%) had mild GIS involvement and 89 (21.1%) had severe GIS involvement. Renal involvement was detected in 171 (21.5%) patients, and was more frequent in those with GIS involvement (26.3% vs. 16.1%, *p* = 0.001). GIS (55.6% (*n* = 306) vs. 47.1% (*n* = 115)) and renal (24.5% (*n* = 134) vs. 15.2% (*n* = 37)) involvement were more common in patients aged > 5 years than in patients ≤ 5 years (*p* = 0.027, *p* = 0.004, respectively). GIS involvement was significantly associated with leukocytosis (*p* < 0.001) and elevated C-reactive protein (CRP) (*p* = 0.018), but these parameters did not correlate with renal involvement. Patients with positive fecal occult blood tests had a significantly higher risk of renal involvement (*p* < 0.001). However, there was no significant difference in renal involvement between patients with mild and severe GIS involvement (*p* = 0.082).

*Conclusion*: GIS involvement, older age (> 5 years), and the presence of occult blood in stool were associated with a higher likelihood of renal involvement in pediatric IgA vasculitis. However, the severity of GIS involvement did not correlate with renal involvement, suggesting that renal pathology may be influenced by independent mechanisms rather than the severity of GIS symptoms.

**Table:** 

**What is Known:** • *Older age, persistent palpable purpura, abdominal pain, GIS involvement, recurrent disease episodes are risk factors for renal involvement in IgA vasculitis.*
**What is New:** • *GIS involvement, fecal occult blood positivity, and age over five years were significantly associated with renal involvement in pediatric IgA vasculitis. However, the severity of GIS involvement did not predict the presence or severity of renal involvement.*

**What is Known:**

• *Older age, persistent palpable purpura, abdominal pain, GIS involvement, recurrent disease episodes are risk factors for renal involvement in IgA vasculitis.*

**What is New:**

• *GIS involvement, fecal occult blood positivity, and age over five years were significantly associated with renal involvement in pediatric IgA vasculitis. However, the severity of GIS involvement did not predict the presence or severity of renal involvement.*

## Introduction

Immunoglobulin A (IgA) vasculitis is the most common systemic vasculitis in childhood, characterized by nonthrombocytopenic palpable purpura, gastrointestinal system (GIS) involvement, arthritis/arthralgia, and renal involvement [1, 2]. The disease is immune complex-mediated, primarily triggered by abnormal IgA1 deposition in small vessels, leading to systemic vasculitis. Although generally self-limiting, with symptoms resolving within an average of four weeks, complications can arise in both the acute and chronic phases. The most frequent acute complications involve the GIS, including intussusception, bowel perforation, and massive GIS bleeding [3, 4].

While GIS complications contribute to short-term morbidity, renal involvement is the primary determinant of long-term morbidity and mortality. Approximately 20% to 50% of pediatric patients develop renal involvement within 4 to 6 weeks following disease onset [5]. The clinical spectrum of renal involvement varies from transient microscopic hematuria and mild proteinuria to severe presentations such as nephrotic syndrome, rapidly progressive glomerulonephritis, and end-stage renal disease. Early prediction of renal involvement is crucial for timely intervention, yet a universally accepted predictive biomarker is lacking [3, 6].

Previous studies have identified several risk factors for renal involvement in IgA vasculitis, including older age, persistent palpable purpura, abdominal pain, recurrent disease episodes, and specific laboratory abnormalities. GIS involvement has also been proposed as a potential risk factor, but the relationship between the severity of GIS involvement and renal outcomes remains unclear [7, 8]. Some studies suggest that severe GIS involvement (e.g., massive bleeding, intussusception) might indicate a more aggressive disease course, while others report no significant association between GIS severity and renal outcomes [9, 10].

Given the inconsistencies in existing data, this study aimed to analyze a large cohort of pediatric IgA vasculitis patients to characterize the demographic and laboratory features of mild and severe GIS involvement, evaluate the association between GIS involvement and renal involvement, and determine whether the severity of GIS involvement predicts renal involvement.

## Materials-methods

This retrospective study included pediatric patients aged 0–18 years who were diagnosed with IgA vasculitis at the pediatric rheumatology clinics of two university hospitals between 1997 and 2024. The diagnosis of IgA vasculitis was based on the Ankara 2008 criteria, endorsed by the European League Against Rheumatism (EULAR), Pediatric Rheumatology International Trials Organization (PRINTO), and Pediatric Rheumatology European Society (PRES) [11].

The study protocol was approved by the Ethics Committee of Düzce University School of Medicine (2024/252) and conducted in accordance with the ethical guidelines of the 1975 Declaration of Helsinki (6 th version, 2008). Given the retrospective nature of the study, informed consent was not required.

### Data collection

Patient data were retrospectively retrieved from electronic medical records and patient files. The recorded variables included demographic information such as age, sex, and age at diagnosis, as well as clinical findings, including palpable purpura, joint involvement, GIS involvement, and renal involvement. Additionally, initial laboratory parameters obtained during hospitalization, such as hemoglobin concentration, white blood cell count, platelet count, erythrocyte sedimentation rate, C-reactive protein levels, complete urinalysis, spot urine protein/creatinine ratio, 24-h urinary protein measurement, and fecal occult blood test results were also collected. CRP levels exceeding 0.5 mg/dL were defined as elevated CRP, a white blood cell count greater than 10,000/mm^3^ was classified as leukocytosis [12], and an ESR greater than 10 mm/hr was considered elevated ESR [13].

### Classification of GIS involvement

GIS involvement was classified based on clinical symptoms and laboratory findings. Patients presenting with abdominal pain, vomiting, or occult blood in the stool were categorized as having mild GIS involvement. Those with melena, hematochezia, or intussusception were classified as having severe GIS involvement [14, 15].

### Definition and classification of renal involvement

Renal involvement was defined based on clinical and laboratory findings observed during the disease course. The presence of microscopic or macroscopic hematuria, proteinuria, renal failure, or hypertension was considered indicative of renal involvement. Microscopic hematuria was defined as the presence of more than five erythrocytes per high-power field in urine sediment examination. Proteinuria was assessed using urine protein/creatinine ratios, with a threshold of greater than 0.2 in children over two years of age and greater than 0.5 in children under two years of age. Additionally, a 24-h urine collection confirming protein excretion exceeding 4 mg/m^2^/hour was considered indicative of proteinuria [16]. Hypertension was defined as systolic or diastolic blood pressure at or above the 95 th percentile for age. Renal failure was defined according to KDIGO criteria. Acute renal failure is defined the presence of any of the following: Increase in serum creatinine by 0.3 mg/dL or more (26.5 μmol/L or more) within 48 h; Increase in serum creatinine to 1.5 times or more than the baseline of the prior 7 days; Urine volume less than 0.5 mL/kg/h for at least 6 h [17].

To assess the severity of renal involvement, the Meadow classification system was used. Stage I was defined as isolated hematuria, while Stage II included hematuria with non-nephrotic proteinuria. Stage III was classified as nephritic syndrome, Stage IV as nephrotic syndrome, and Stage V as nephritic-nephrotic syndrome. For statistical analysis, Stages I and II were considered mild renal involvement, whereas Stages III, IV, and V were classified as severe renal involvement [18, 19].

### Bias and data validation

To minimize selection bias, all eligible patients diagnosed with IgA vasculitis during the study period were included. Data accuracy was ensured by cross-checking patient records with electronic medical records, and two independent researchers verified the consistency of the collected data. A total of 87 patients were excluded due to missing key clinical variables such as anamnesis, clinical findings, physical examination findings, laboratory findings (complete urinalysis, fecal occult blood test, 24-h urinary protein measurement) which we used to define renal and GIS involvement.

## Statistical analyses

Statistical analyses were conducted using IBM SPSS v.22 software. The distribution of numerical variables was assessed using the Kolmogorov–Smirnov test to determine normality. Descriptive statistics are presented as means and standard deviations for normally distributed variables, whereas non-normally distributed variables are reported as medians with interquartile ranges or minimum–maximum values. Group comparisons for normally distributed continuous variables were performed using independent samples t-tests, while non-normally distributed continuous variables were analyzed using the Mann–Whitney U test. Categorical variables are presented as frequencies and percentages. The relationships between categorical variables were analyzed using Pearson’s chi-square test, Fisher’s exact test, or the Fisher–Freeman–Halton test, depending on the expected value distribution and the number of groups. Multiple logistic regression analyses were conducted to identify significant factors independently associated with GIS and renal involvement. Variables were initially assessed through univariate analyses, and statistically significant variables were subsequently included in the multiple model to verify whether the factors found to be significantly associated with involvement remain significant after correcting for potential confounders. A *p*-value of < 0.05 was considered statistically significant.

## Results

A total of 87 patients were excluded due to lack of data. A total of 794 patients were included in the study, of whom 430 (54.2%) were male (54.2%). The mean age at diagnosis was 7.8 ± 3.3 years, with similar distributions between females (7.8 ± 3 years) and males (7.8 ± 3.4 years). The majority of patients (69.2%) were older than five years. The demographic characteristics and clinical findings of the patients are presented in Table 1.

**Table 1:** Demographic characteristics and clinical findings of patients with IgA vasculitis

Category	n (%)
**Age**	
≤ 5 years	244 (30.8)
> 5 years	550 (69.2)
**Sex**	
Female	364 (45.8)
Male	430 (54.2)
**Systemic Involvements**	
Palpable Purpura	794 (100)
Gastrointestinal Involvement	422 (53.1)
Mild GIS Involvement	333 (42)
Severe GIS Involvement	89 (11.1)
Abdominal Pain	343 (43.1)
Vomiting	46 (5.9)
Occult Stool Blood	274 (34.5)
Hematochezia/Melena	76 (9.5)
Intussusception	13 (1.6)
Renal Involvement	171 (21.5)
Mild Renal Involvement	87 (10.9)
Severe Renal Involvement	84 (10.6)
Joint Involvement	351 (44.2)

GIS involvement was observed in 422 patients (53.1%), with 333 (42%) classified as mild GIS involvement and 89 (11.1%) as severe GIS involvement. The most common GIS symptoms were abdominal pain (43.1%), vomiting (5.9%), and occult stool blood positivity (34.5%). Severe GIS involvement included hematochezia/melena (9.5%) and intussusception (1.6%). Renal involvement was identified in 171 patients (21.5%), with 87 (10.9%) classified as mild and 84 (10.6%) as severe renal involvement. Among these patients, 20 (2.5%) had nephrotic-range proteinuria, and 64 (8%) had nephritic-range proteinuria.

A comparison of clinical and laboratory findings between patients with and without GIS involvement is presented in Table 2.

**Table 2:** Clinical and laboratory findings in patients with and without GIS involvement

Findings	Non-GIS	GIS	*p*-value
Elevated CRP (> 0.5 mg/dL)	171 (66.3%)	205 (75.6%)	0.018
Leukocytosis (> 10,000/mm^3^)	154 (42.4%)	237 (57.5%)	< 0.001
Elevated ESR (> 10 mm/hr)	247 (90.1%)	300 (90%)	0.982
Proteinuria	27 (7.3%)	70 (16.6%)	< 0.001
Hematuria	37 (9,9%)	61 (14.5%)	0.065
Renal Involvement	60 (16.1%)	111 (26.3%)	0.001
Occult Stool Blood	0 (0.0%)	274 (64.9%)	< 0.001
> 5 years of age	242 (65%)	306 (72,5)	0,027

*GIS *gastrointestinal system, *CRP *C-reactive protein, *ESR *erythrocyte sedimentation rate, percentages for each laboratory parameter were calculated based on the number of patients with a value of valid measurement

The rates of GIS and renal involvement did not significantly differ between males and females (*p* = 0.075, *p* = 0.781, respectively). Additionally, no significant difference was found between mild and severe GIS involvement based on sex (*p* = 0.444).

When analyzed by age groups, GIS involvement was more common in patients older than five years (55.6% in > 5 years vs. 47.1% in ≤ 5 years, *p* = 0.027). Similarly, renal involvement was significantly more frequent in the > 5 years group (24.5% vs. 15.2%, *p* = 0.004). Among those with GIS involvement, renal involvement was significantly more frequent in the > 5 years group (*p* = 0.042). However, no significant difference was found between mild and severe GIS involvement in predicting renal involvement (*p* = 0.084).

A comparison of clinical and laboratory findings between patients with and without renal involvement is presented in Table 3.

**Table 3:** Clinical and laboratory findings in patients with and without renal involvement

Findings	Without Renal	Renal	*p*-value
Elevated CRP (> 0.5 mg/dL)	288 (71.1%)	88 (71%)	0.975
Leukocytosis (> 10,000/mm^3^)	310 (51.2%)	81 (47.9%)	0.458
Elevated ESR (> 10 mm/hr)	429 (91.3%)	118 (86.1%)	0.076
GIS involvement	311 (49.9%)	111 (64.9%)	0.001
Severe GIS involvement	72 (11.6%)	17 (9.9%)	0.082
Mild GIS involvement	239 (38.4%)	94 (54.9%)	0.082
Occult Stool Blood	193 (31%)	81 (47.4%)	< 0.001
> 5 years of age	414 (66.4%)	134 (78.4%)	0.004

*GIS *gastrointestinal system, *CRP *C-reactive protein, *ESR *erythrocyte sedimentation rate, percentages for each laboratory parameter were calculated based on the number of patients with a value of valid measurement

**Fig. 1:**
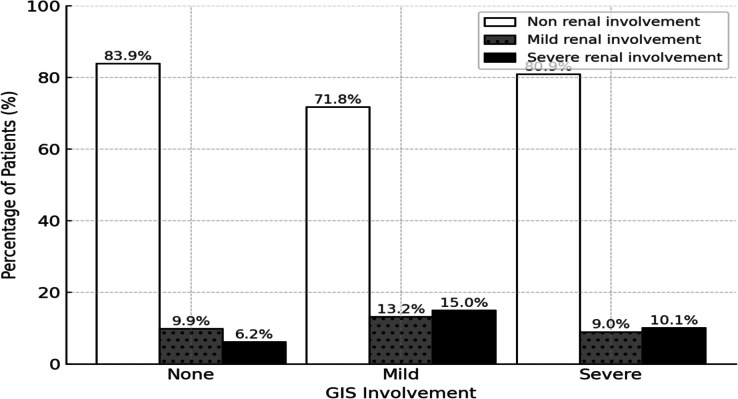
Association of GIS involvement with kidney involvement

Figure 1 illustrates the distribution of renal involvement among patients with different levels of GIS involvement. The bars represent the proportion of patients with no renal involvement, mild renal involvement, and severe renal involvement across three GIS categories (none, mild, and severe involvement). Patients with GIS involvement had a higher rate of renal involvement, but the severity of GIS involvement did not show a significant correlation with renal involvement severity.

As a result of the multiple logistic regression analysis, including the variables that were found significantly associated in the univariate analyses initially, CRP > 0.5 mg/dL (odds ratio (OR): 1.595, 95% confidence interval (CI): 1.090–2.335, *p* = 0.016), leukocytosis > 10,000/mm^3^ (OR: 1.952, 95% CI: 1.459–2.610, *p* < 0.001), and proteinuria (OR: 2.669, 95% CI: 1.507–4.726, *p* = 0.001) were found significantly associated with GIS involvement (Table 4). On the other hand, the multiple logistic regression analysis revealed that GIS involvement (OR: 1.978, 95% CI: 1.083–3.613, *p* = 0.026) and occult stool blood (OR: 1.817, 95% CI: 1.100–3.003, *p* = 0.019), age above five years (OR: 1.62; 95% CI: 1.11–2.36; *p* = 0.012), were found significantly associated with renal involvement at the final optimal model (Table 5).

**Table 4:** Logistic regression analysis results for the factors associated with GIS involvement

Findings	OR	95% CI	*p*-value
Elevated CRP (> 0.5 mg/dL)	1.595	1.090—2.335	0.016
Leukocytosis (> 10,000/mm^3^)	1.952	1.459- 2.610	< 0.001
Proteinuria	2.669	1.507—4.726	0.001

*GIS *gastrointestinal system, *CRP *C-reactive protein, *OR *odds ratio, *CI *confidence interval, χ^2^ = 24.581, *p* < 0.001, Hosmer–Lemeshow *p* = 0.948

**Table 5:** Logistic regression analysis results for the factors associated with renal involvement

Findings	OR	95% CI	*p*-value
GIS involvement	1.978	1.083—3.613	0.026
Occult Stool Blood	1.817	1.100—3.003	0.019
> 5 years of age	1,62	1.11–2.36	0.012

*GIS *gastrointestinal system, *OR *odds ratio, *CI *confidence interval, χ^2^ = 8.611, *p* = 0.014, Hosmer–Lemeshow *p* = 0.939

## Discussion

In this study, GIS involvement was observed in 53.1% of pediatric patients with IgA vasculitis, with 42% classified as mild and 11.1% as severe. These findings are in line with previous studies [14, 19], where GIS involvement rates range between 50 and 75%, though variations exist in the proportion of mild and severe cases. Karadağ et al. [15] reported GIS involvement in 51.3% of patients, with 38.4% classified as mild and 12.9% as severe, whereas Li et al. [14] found GIS involvement in 50%, with 24.4% having severe GIS involvement. Several factors have been associated with severe GIS involvement, including early or late onset age (< 3 years or 13–17 years), purpura on the trunk, vomiting, a high neutrophil-to-lymphocyte ratio, and decreased serum albumin levels. Additionally, severe abdominal pain and GIS symptoms appearing before the onset of palpable purpura have been proposed as risk factors.

Compared to previous reports [14, 20], our study found a lower prevalence of severe GIS involvement (11.1%), particularly in contrast to studies from China, where severe cases have been reported at higher rates. This discrepancy may be attributed to genetic and geographical differences, as well as differences in referral patterns, given that our study was conducted at a tertiary referral center, where milder cases may not always be admitted. Furthermore, GIS involvement was more frequently observed in patients older than five years, reinforcing findings from prior research. However, we found no significant difference between males and females regarding the severity of GIS involvement.

Laboratory findings indicated that GIS involvement was more common in patients with leukocytosis and elevated CRP levels, suggesting a systemic inflammatory response in these patients. Proteinuria was found significantly associated with GIS involvement. However, no significant associations were found between GIS involvement and ESR, hemoglobin levels, or platelet counts. Additionally, the severity of GIS involvement did not correlate with significant variations in laboratory parameters, indicating that inflammation alone may not fully explain the severity of GIS symptoms [21].

Renal involvement is the most critical determinant of long-term morbidity and mortality in IgA vasculitis, with prevalence rates ranging from 20 to 80% [22, 23]. The wide variation in reported renal involvement rates is attributed to differences in ethnicity, geographic region, study design, clinical settings, and follow-up duration. Studies have indicated that renal involvement develops within four weeks in 85% of cases, within six weeks in 91%, and within six months in 97% [6, 24]. In this study, renal involvement was observed in 21.5% of patients, aligning with the lower end of the reported range. This relatively low rate of renal involvement may be due to early referral to pediatric rheumatology clinics and potential underdiagnosis of subclinical renal involvement due to the retrospective nature of the study.

Early identification of nephritis is crucial to preventing progression to end-stage renal disease. Several studies have proposed various risk factors for renal involvement, including older age, persistent purpura, abdominal pain, and elevated inflammatory markers. Severe GIS bleeding, relapses, and atypical purpura distribution have also been associated with an increased risk of nephritis. However, there is no universally accepted age threshold for predicting renal involvement, with different studies suggesting cutoffs at four, six, or eight years [19, 25]. In this study, renal involvement was more frequently observed in patients older than five years, further supporting the notion that older age is a potential risk factor for renal complications.

A meta-analysis identified male sex as a potential risk factor for nephritis, but in this study, no significant sex-related differences in renal involvement were found [19]. The same meta-analysis also suggested that severe GIS involvement might increase the risk of renal complications. However, in this study, although GIS involvement itself was significantly associated with renal involvement, there was no difference between mild and severe GIS involvement in predicting renal complications. Additionally, no correlation was found between the severity of GIS involvement and the severity of renal involvement, suggesting that GIS and renal involvement may develop through independent pathophysiological mechanisms rather than being directly linked.

Previous studies have investigated potential laboratory predictors of renal involvement in IgA vasculitis [7, 24]. One study identified female sex and an elevated neutrophil-to-lymphocyte ratio as risk factors for renal involvement, but no significant differences in WBC count, hemoglobin concentration, platelet count, or CRP levels were found between patients with and without renal involvement [25]. Similarly, another studies found that age over six years, purpura in atypical locations, and positive fecal occult blood tests were associated with nephritis, whereas WBC count, hemoglobin concentration, and CRP levels did not significantly predict renal involvement [26, 27]. In the present study, renal involvement was significantly more common in patients with positive fecal occult blood tests, whereas no significant associations were observed between renal involvement and leukocytosis, ESR, hemoglobin levels, platelet counts, or CRP levels.

This study has several strengths, including the large sample size of 794 pediatric patients, making it one of the most comprehensive investigations of GIS and renal involvement in IgA vasculitis. However, several limitations should be acknowledged. The retrospective nature of the study may have introduced selection bias and incomplete data collection. Additionally, the long study period spanning 27 years may have led to variability in diagnostic and treatment approaches, potentially affecting the consistency of findings. Another limitation is the absence of renal biopsy data, which restricts the ability to correlate clinical and laboratory findings with histopathological outcomes. Furthermore, genetic and environmental factors influencing disease severity were not assessed.

Future studies should focus on prospective, multi-center investigations with standardized diagnostic criteria and longer follow-up periods to validate the observed associations. To improve early risk stratification for nephritis in pediatric IgA vasculitis, findings from renal biopsies should be evaluated and the potential role of urinary cytokine profiles and urinary metabolites should also be investigated.

## Conclusion

This study demonstrated that GIS involvement, fecal occult blood positivity, and age over five years were significantly associated with renal involvement in pediatric IgA vasculitis. However, the severity of GIS involvement did not predict the presence or severity of renal involvement, suggesting that GIS and renal complications may arise through distinct mechanisms. These findings highlight the importance of early screening for renal involvement in patients with GIS symptoms, regardless of severity, and emphasize the need for further research into the underlying pathophysiology of IgA vasculitis.

## Data Availability

No datasets were generated or analysed during the current study.

## Abbreviations

Ig A: Immunoglobulin A
GIS: Gastrointestinal system
ESR: Erythrocyte sedimentation rate
CRP: C-reactive protein
